# Deep Compartment Syndrome Without Myonecrosis: A Case Report on a Rare Complication of Sickle Cell Disease

**DOI:** 10.7759/cureus.29164

**Published:** 2022-09-14

**Authors:** Per O Iversen, Alexandra Hankin, Joachim Horn, Torkild H Pedersen, Ruth Borgersen, Hege M Frøen

**Affiliations:** 1 Department of Haematology, Oslo University Hospital, Oslo, NOR; 2 Department of Nutrition, University of Oslo, Oslo, NOR; 3 Department of Haematology and Blood Transfusion,, Muhimbili University of Health and Allied Sciences, Dar es Salaam, TZA; 4 Department of Radiology, Oslo University Hospital, Oslo, NOR; 5 Division of Orthopaedic Surgery, Oslo University Hospital, Oslo, NOR

**Keywords:** rhabdomyolysis, vaso occlusive crisis, sickle cell disease complications, aseptic myonecrosis, compartment syndrome leg

## Abstract

Compartment syndrome is a rare manifestation of vaso-occlusive crisis, a serious complication of sickle cell disease (SCD), which is an inherited hemoglobinopathy. During a visit to Norway, an otherwise healthy, 20-year-old male from Ghana was admitted to Oslo University Hospital (Day 1) because of increasing pain in the hip and thighs that did not respond adequately to non-opioid painkillers. Despite initial treatment with intravenous fluids and opioids, his pain intensified. Careful clinical inspection supported by an MRI examination revealed focal, high-signal-intensity muscle edema of the anterior compartment of the thigh, almost exclusively limited to the vastus intermedius muscles. There were no MRI findings or blood biochemistry evidence for myonecrosis or rhabdomyolysis, and a diagnosis of deep compartment syndrome appeared to be the most likely explanation for his pain. We decided to continue with a conservative treatment approach, and the patient did not undergo a fasciotomy or blood transfusion therapy. On Day 7 after admission, his condition improved markedly, and he was discharged on Day 11 whereupon he returned to Ghana. This case is a reminder that, although rare, deep compartment syndrome can be a severe manifestation of vaso-occlusive crisis in SCD and should be considered in patients with severe, deep muscular pain in the absence of other explanatory factors.

## Introduction

Sickle cell disease (SCD) is an inherited recessive disorder affecting the production of hemoglobin (Hb) [1]. In SCD patients, the polymerization of Hb molecules, usually due to lowered oxygen tension, alters the physiochemical properties of the erythrocytes so they become rigid and short-lived, in addition to forming microthrombi that block capillaries and thereby impeding blood flow. Erythrocytes undergoing such “sickling” also adhere to the underlying vascular lining, thus changing vascular permeability as well as creating local inflammation [2]. These pathological processes explain the chronic hemolysis and the recurrent vaso-occlusive crisis (VOC) that these patients experience as pain, usually in the bones and skeletal muscles. The distribution of SCD is most prevalent among populations residing around the equatorial belt, in particular sub-Saharan Africa [3].

Compartment syndrome of the superficial part of skeletal muscles in the extremities is a rare complication of VOC in SCD, often leading to myonecrosis, and has been described among both homozygous (HbSS) and combined heterozygous (e.g. HbSC) SCD patients [4-6]. Rhabdomyolysis has often been proposed as the precipitating factor of VOC-related compartment syndrome [6,7]. Here, we present an unusual case of deep compartment syndrome without signs of myonecrosis or rhabdomyolysis in the thighs of an otherwise healthy African male with HbSS.

## Case presentation

In July 2022, a male soccer trainer from Ghana, aged 20 years, arrived in Sweden for a sporting event. He was diagnosed with HbSS as a child but had a few episodes of VOC, the last being in 2016, which required hospitalization and transfusion of one unit of packed red blood cells. His habitual Hb was between 9 and 10 g/dl. He had no other acute or chronic disease and only used folic acid (1 mg daily).

A few days prior to his departure from Ghana, he had experienced mild symptoms of an upper respiratory tract infection but without fever or cough. After a few days in Sweden, he started to experience bone pain. He visited an outpatient clinic and was prescribed a non-steroid anti-inflammatory drug to only moderate effect. A few days later, he arrived in Norway for a similar sporting event. His pain had escalated to a score of 8/10 on the Numeric Rating Scale, and he was admitted to our ward under suspicion of VOC (Day 1). He presented with severe lower back pain as well as severe pain in both thighs. On admission, his Hb was 8.0 g/dl, HbS fraction 94%, HbF fraction 1%, HbA not detected, thrombocytes 563 x 10^9^/l, leukocytes 20.5 x 10^9^/l (dominated by mature neutrophilic granulocytes), LDH 730 U/l, haptoglobin not detected, creatinine kinase (CK) 42 U/l, bilirubin 26 micromol/l, creatinine 53 micromol/l, and C-reactive protein (CRP) 348 mg/l. He tested negative for a polymerase chain reaction (PCR)-based coronavirus disease 2019 (COVID-19) test, no malaria parasites were detected on a blood smear, and his urine dipstick was negative, as was his chest X-ray.

Treatment was initiated with intravenous saline (2-3 l per day), intravenous opioids (oxycodone, 50-60 mg per day (Days 1-5, then gradual reduction on Days 6-10), and thromboprophylaxis with subcutaneous dabigatran 5000 U/day. He also received oral paracetamol (1 g 4 times per day). On Day 3, fever erupted (rectal temperature of 39 ^o ^C). We then initiated antibiotic treatment with intravenous meropenem (2 g three times daily). Later, a throat specimen revealed that he was infected with methicillin-resistant Staphylococcus aureus bacteria, although it was unclear if this had any pathogenic role in this setting.

**Figure 1:**
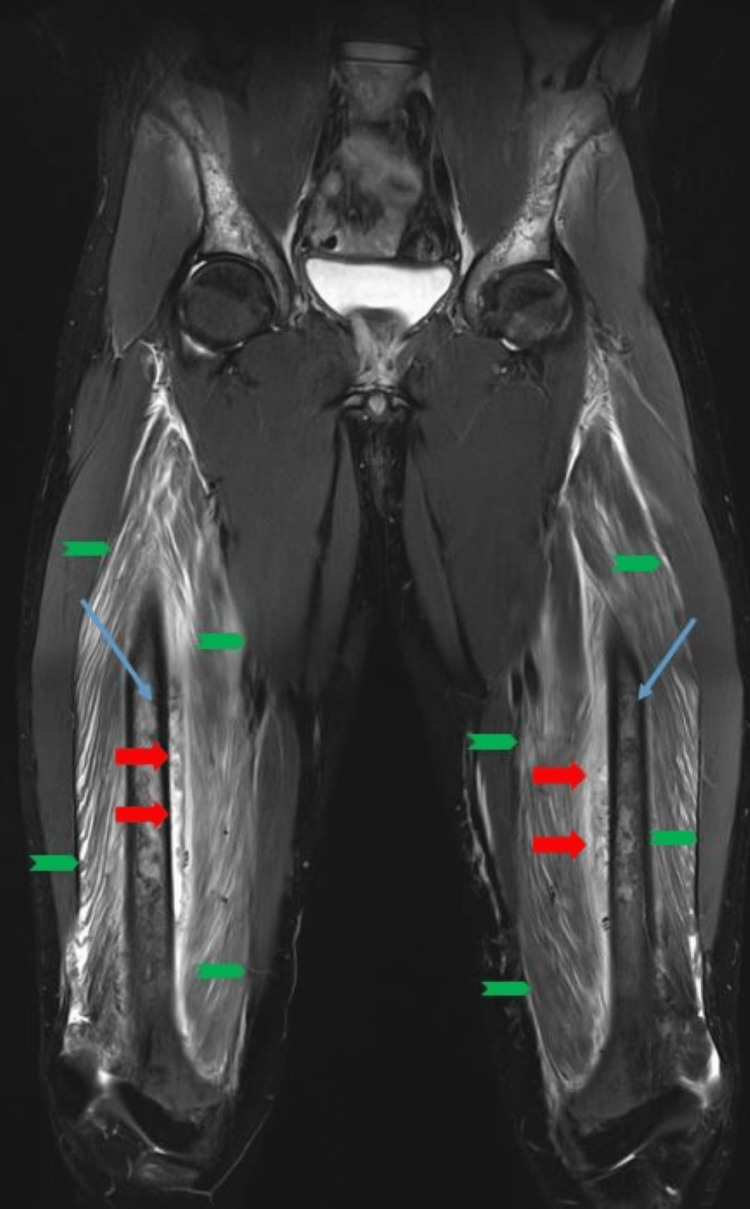
Day 6 coronal MRI scan A coronal T2 short inversion time inversion (STIR) image (Siemens 3T MRI scanner; Munich, Germany) was taken of the lower limbs and pelvis from approximately 5 cm above the iliac crest to approximately 10 cm below the knee joints on Day 6 after hospital admission. The image showed focal, high signal intensity muscle edema of the anterior compartment of the thigh, almost exclusively limited to the vastus intermedius muscle with minimal affection of the vastus medialis and biceps femoris (green arrows). Multiple bone infarcts (blue arrow) were seen in both the femur diaphysis as well as the iliac bones on both sides. There was also a small amount of muscle edema seen in the muscles around the iliac bones. In addition, there was significant periosteal lifting with associated fluid signal (red arrows) medially along both femur shafts where the infarcts had occurred in the thighs.

**Figure 2:**
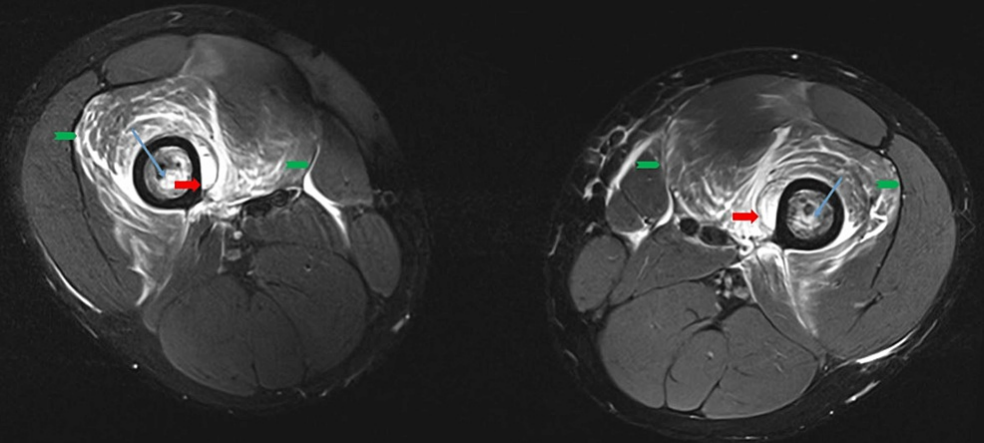
Day 6 axial MRI scan An axial T1 turbo spin echo (TSE) MRI image was taken of the lower limbs on Day 6 after hospital admission. The image on the day showed focal, high signal intensity muscle edema of the anterior compartment of the thigh, almost exclusively limited to the vastus intermedius muscle with minimal affection of the vastus medialis and biceps femoris (green arrows). Bone infarcts (blue arrow) were seen in both femur diaphysis. In addition, there was significant periosteal lifting with associated fluid signal (red arrows) medially along both femur shafts where the infarcts had occurred in the thighs.

Careful palpation revealed no apparent increased pressure in the superficial muscles. However, more forceful palpation of the thighs was perceived as very painful. On suspicion of threatening compartment syndrome, we performed MRI scans of his hip and thighs on Day 6 (Figures 1, 2). While infection or osteomyelitis could not be excluded based on these MR images alone, taken in conjunction with the clinical findings, a deep muscle compartment syndrome was considered the most likely diagnosis.

**Figure 3:**
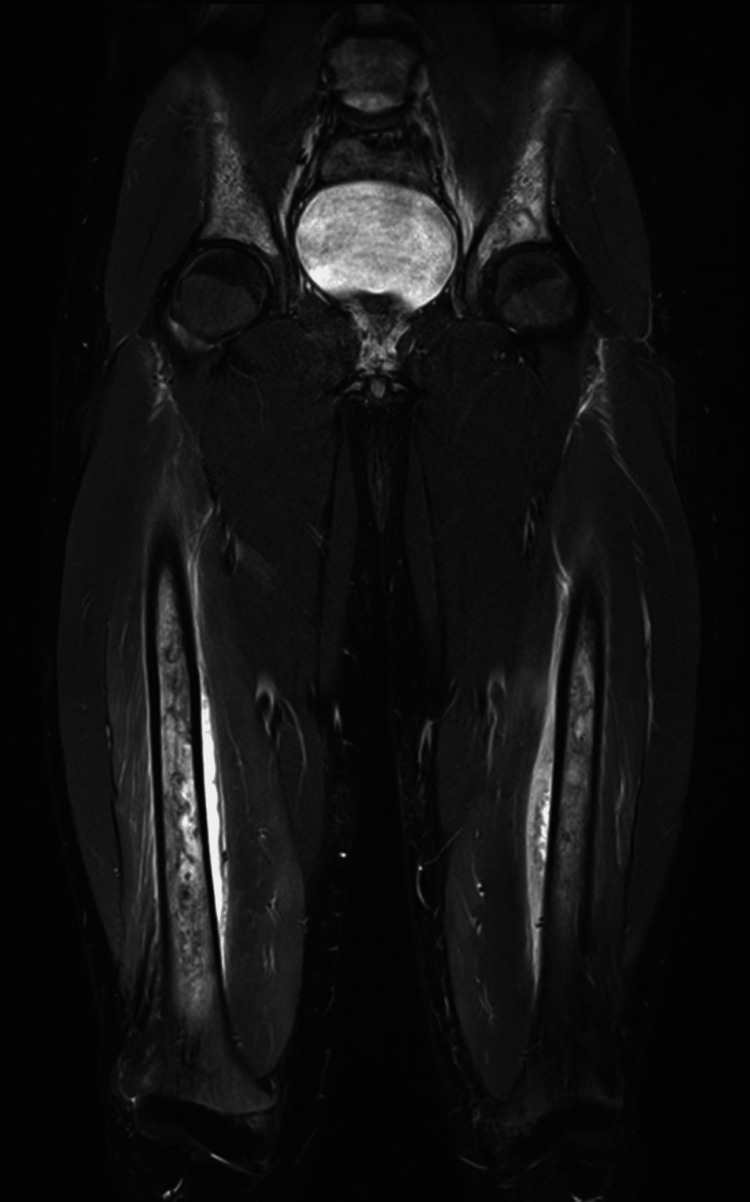
Day 10 coronal MRI scan A coronal T2 short inversion time inversion (STIR) image was taken on Day 10 after hospital admission. The image showed a significant reduction in muscle edema compared to the image taken on Day 6, however, the periosteal lifting and bone infarctions remained essentially unchanged.

**Figure 4:**
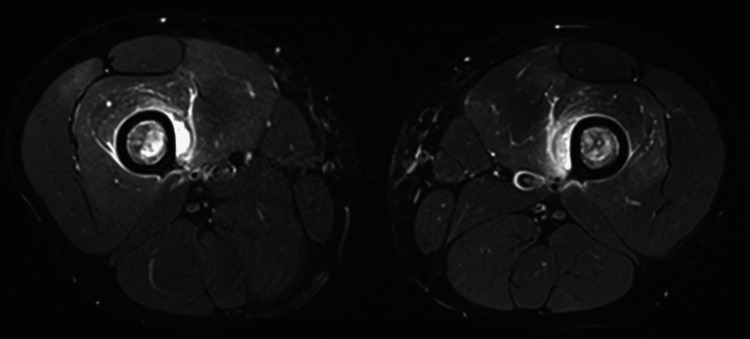
Day 10 axial MRI scan An axial T1 turbo spin echo (TSE) MRI image was taken of the lower limbs on Day 10 after hospital admission. The image showed a significant reduction in muscle edema compared with the Day 6 image, however, the periosteal lifting and bone infarctions remained essentially unchanged.

Both surgical intervention and exchange blood transfusion were considered as the next therapeutic steps, but within the next 24 hours, his condition improved. On Day 10, follow-up MRI scans showed a significant reduction in both the intensity and distribution of the muscle edema (Figures 3, 4). The focal periosteal findings and multiple bone infarctions remained unchanged. Due to a pre-booked return flight to Ghana, he was discharged from our hospital on Day 11. He then felt better, and most of his blood values had improved: Hb 7.8 g/dl, thrombocytes 1180 x 10^9^/l, leukocytes 14.4 x 10^9^/l, lactate dehydrogenase (LDH) 459 U/l, CK 95 U/l, myoglobin 23 microg/l, bilirubin 14 micromol/l, creatinine 42 micromol/l, and CRP 116 mg/l. A week after returning to Ghana, the first author was in contact with him, and he felt fine with no discomfort, in particular no bone or muscle pain.

## Discussion

The previous upper airway infection and/or changing environment from hot Ghana (about 30 ^o^C) to a chillier Scandinavia (about 17 ^o^C) [8] may have triggered the VOC in this otherwise healthy SCD patient. Before the current VOC, he had been little affected by the complications of his SCD; the last hospitalization was back in 2016. Even though his complaints were less severe back then, the pain was still in the same place as it was now, in his hips and thighs.

Compartment syndrome as part of a VOC in SCD is reportedly rare [7,9]. However, as it can be difficult to diagnose, its prevalence may have been underestimated. The deep compartment syndrome in our patient was diagnosed by a clinical examination by a specialist in orthopedic surgery combined with digital imaging (MRI) of the thighs. The absence of myonecrosis was based on the CK and myoglobin blood results, which were normal, and the MRI findings, which did not suggest myonecrosis. Thus, rhabdomyolysis probably did not occur. A biopsy was not collected from the affected tissue (deep in the thigh close to the bone), as this would have been unlikely to have changed the choice of treatment.

In severe cases of compartment syndrome, surgical intervention may be necessary to prevent muscle necrosis and the subsequent development of muscular atrophy and contractures, and can even be life-saving. In our case, we chose a conservative approach as his condition showed signs of improvement a week after admission to our ward. For the same reason, we decided not to give him a blood transfusion, and his Hb remained stable (7-8 g/dl) throughout the hospitalization.

It is unclear why some SCD patients develop compartment syndrome as part of VOC. It has been suggested that patients with the compound HbSC genotype are more susceptible to developing this complication since the combination of several abnormal genes (e.g. HbS and HbC) is more likely to distort intramuscular fluid shifts [4,7]. However, in the few SCD cases reported so far with compartment syndrome, the compound heterozygotes (e.g. HbSC) were not more affected than the homozygous (HbSS) SCD patients [4]. In addition, in their comprehensive review of skeletal muscle manifestations in SCD, Merlet et al. also did a thorough review of the skeletal manifestations of SCD and found that most of the pathological findings were caused by the HbS gene product [10].

## Conclusions

Compartment syndrome, in particular the deep type, is a rare complication of VOC in patients with SCD. It is important to diagnose this complication in order to prevent muscular atrophy and contractures, which may lead to future mobility disability. Careful clinical examination in conjunction with relevant blood work-up and supported by MRI imaging, are important tools to assist in making this diagnosis. A conservative treatment approach with intravenous fluids and pain relief may be sufficient, but the patients should be carefully monitored to identify cases in which surgery and/or blood transfusion are required.
